# Supplementary material for: Selfish migrants: How a meiotic driver is selected to increase dispersal
Source: J Evol Biol. 2022 Mar 7;35(4):621–32. doi: 10.1111/jeb.13989 (PMC9311743; doi:10.1111/jeb.13989)
Supplement: Supplementary file 2 — Fig S1‐S10 [file JEB-35-621-s001.pdf]

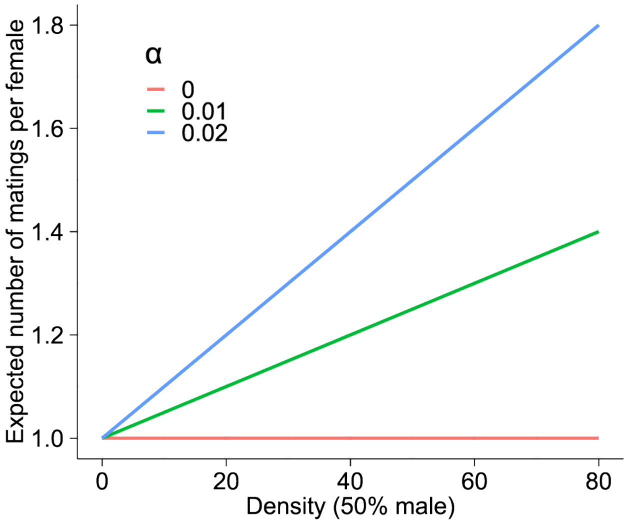

Suppl. Fig. 1: Expected number of matings per female with increasing density at different α values.

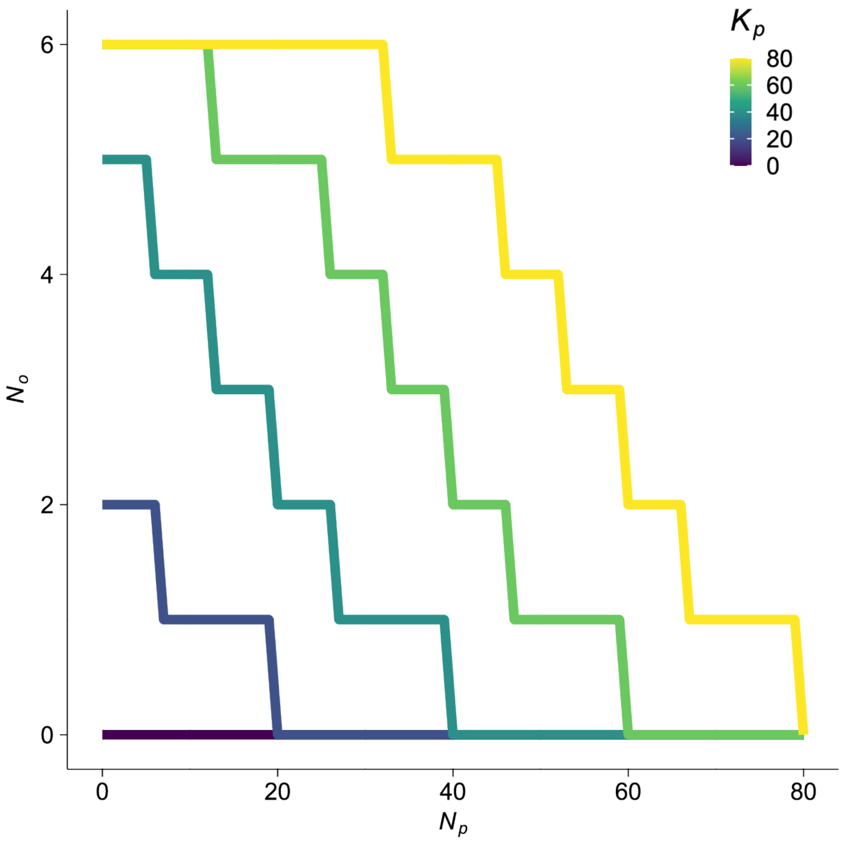

Suppl. Fig. 2: Female fecundity at different densities and carrying capacities.

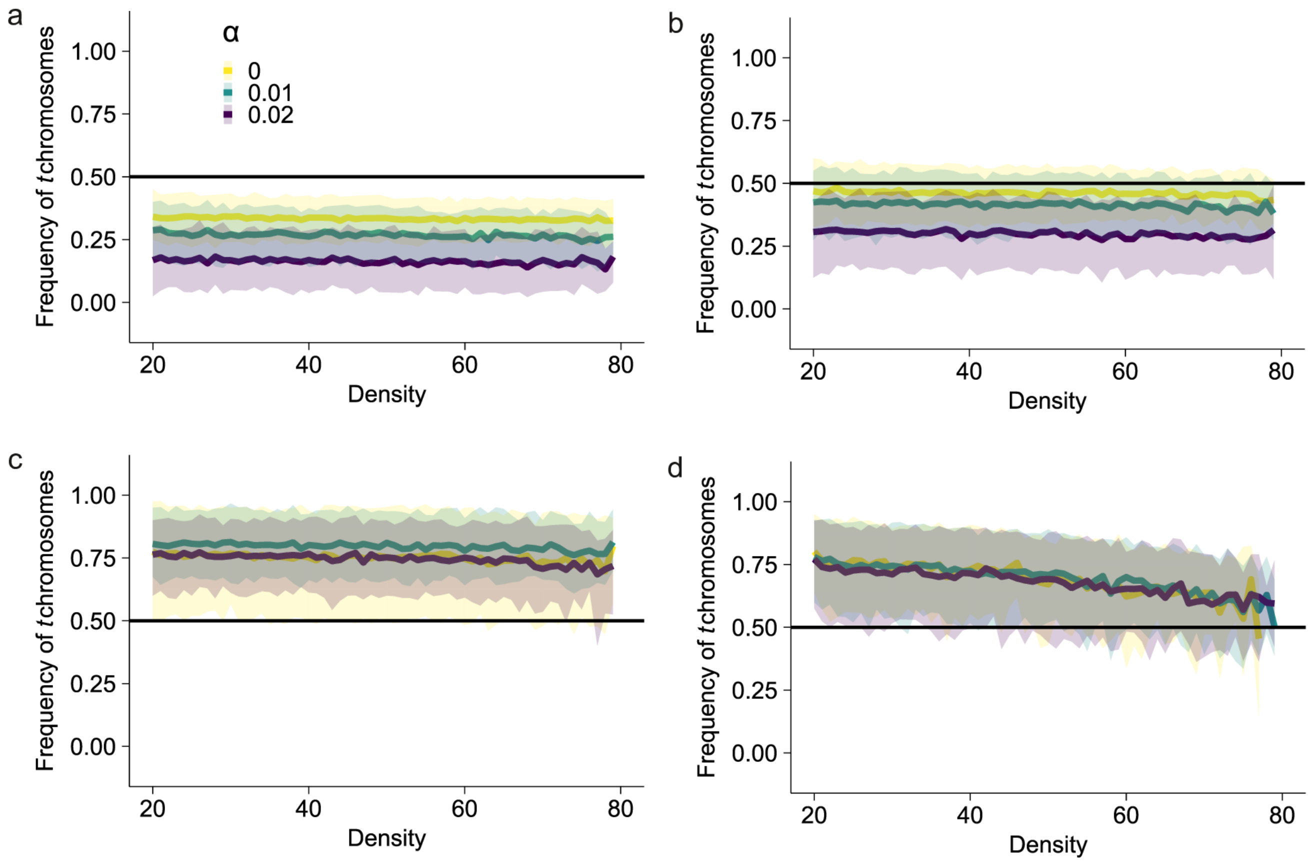

Suppl. Fig. 3: Frequency of t chromosomes (0.5 in $+/t$, 1 in t/t) in simulations after 10,000 turns of t and $+$ coexistence with a fixed dispersal propensity of 0.1 and varying α under $\tau = 0.0$ (a), $\tau = 0.25$ (b), $\tau = 0.50$ (c), or infertile male t/t (d, $\tau = 1$). Lines indicate the mean and ribbons show the standard deviation.

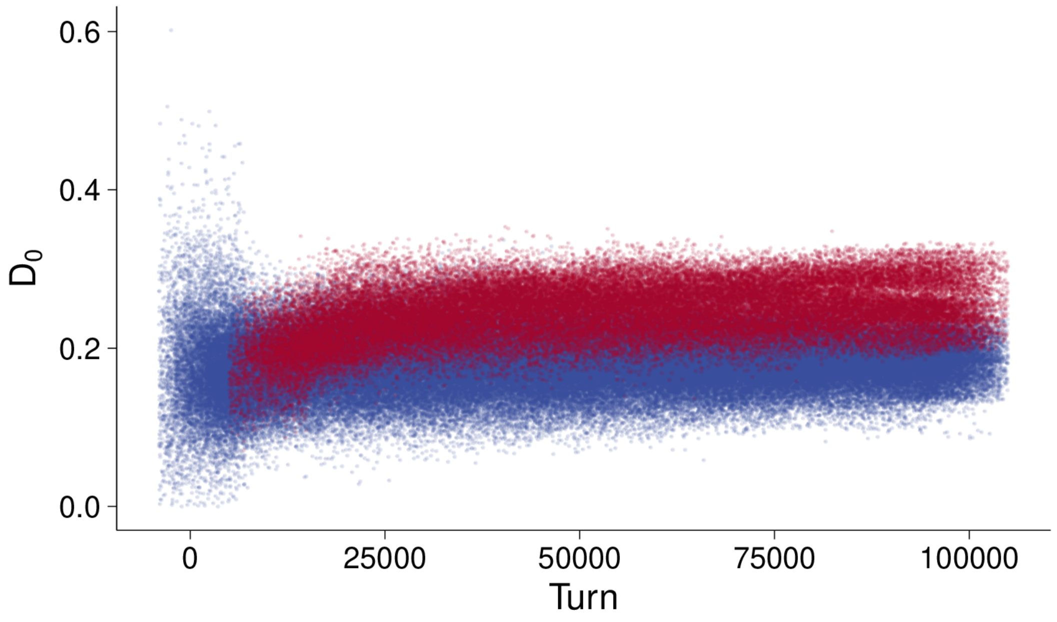

Suppl. Fig. 4: Evolution of dispersal propensity D_0 over the course of the simulation under natural conditions with one locus. A random subset of individual values is visible as dots, jittered horizontally. t values are in red, $+$ values are in blue.

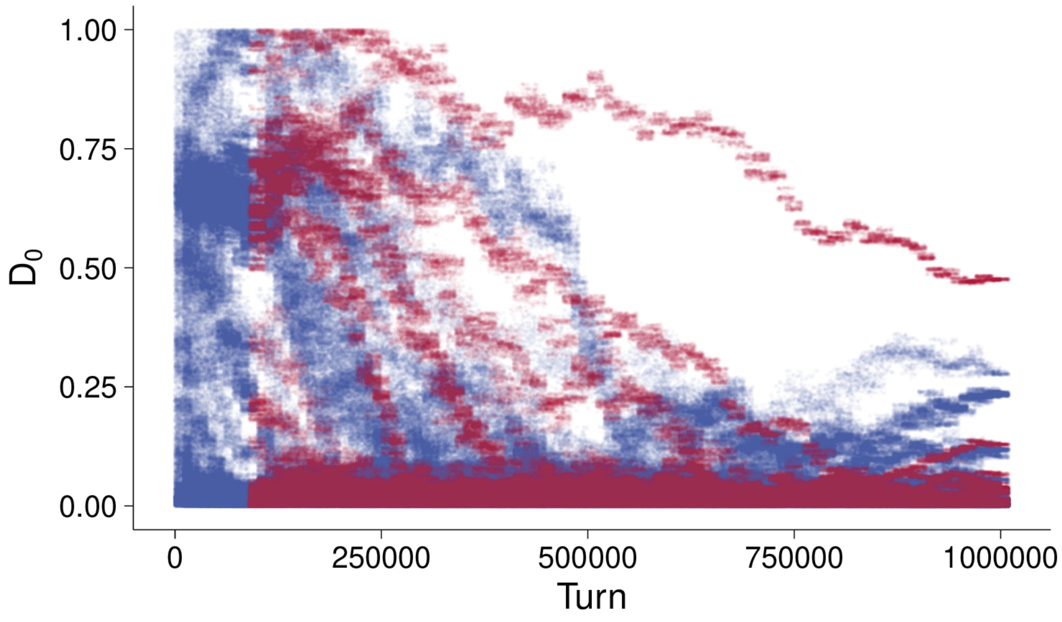

Suppl. Fig. 5: Evolution of dispersal propensity intercept D_0 over the course of the simulation under natural conditions (two-locus models). A random subset of individual values is visible as dots, jittered horizontally. t values are in red, $+$ values are in blue.

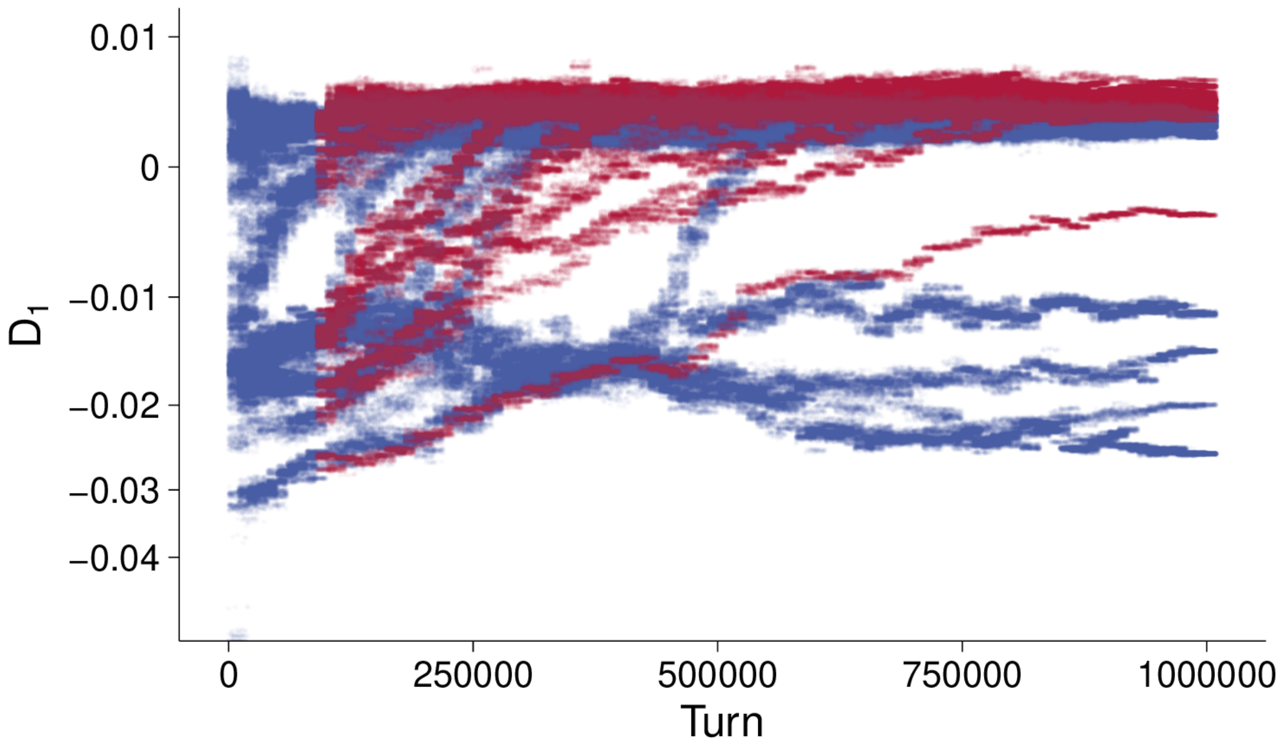

Suppl. Fig. 6: Evolution of dispersal propensity slope D_1 over the course of the simulation under natural conditions (two-locus models). A random subset of individual values is visible as dots, jittered horizontally. t values are in red, + values are in blue.

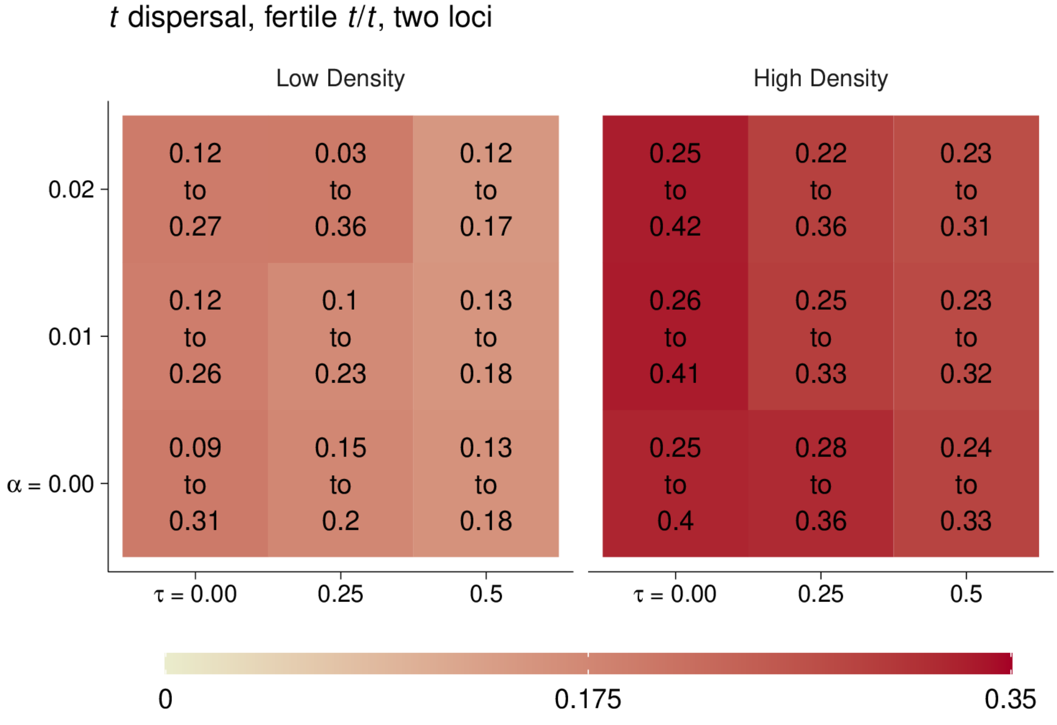

Suppl. Fig. 7: Heatmap showing the mean dispersal propensity of t in two-locus models in low and high densities in varying polyandry α and t/t viability τ. Red indicates increased t dispersal. The text indicates the 95% confidence interval.

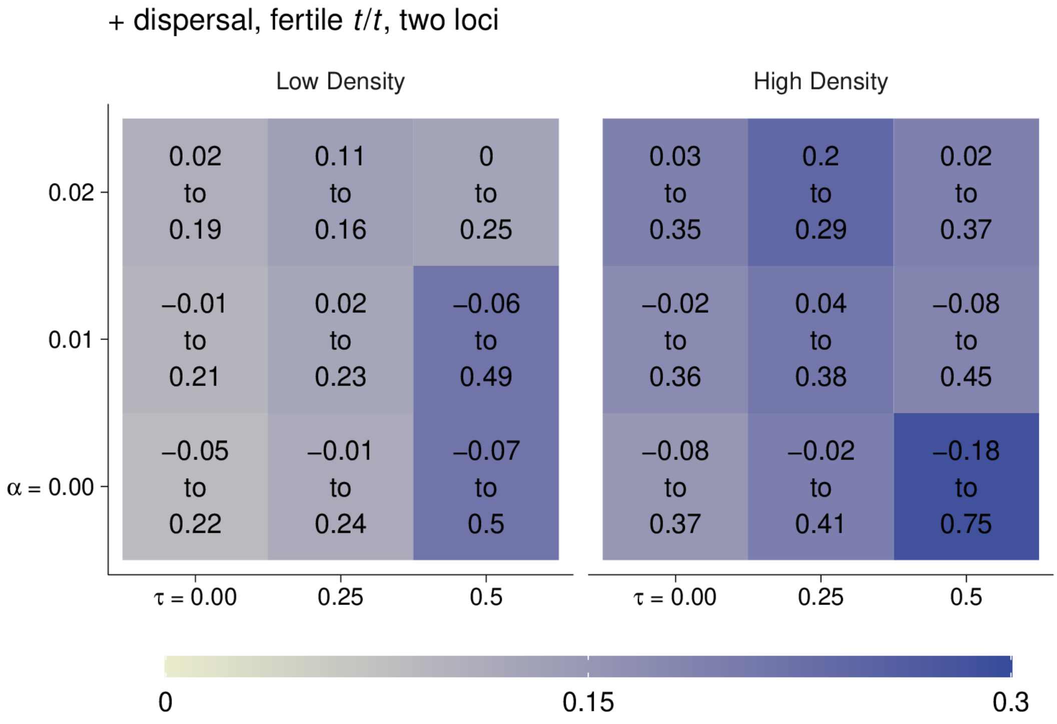

Suppl. Fig. 8: Heatmap showing the mean dispersal propensity of + in two-locus models in low and high densities in varying polyandry α and t/t viability τ. Blue indicates increased + dispersal. The text indicates the 95% confidence interval.

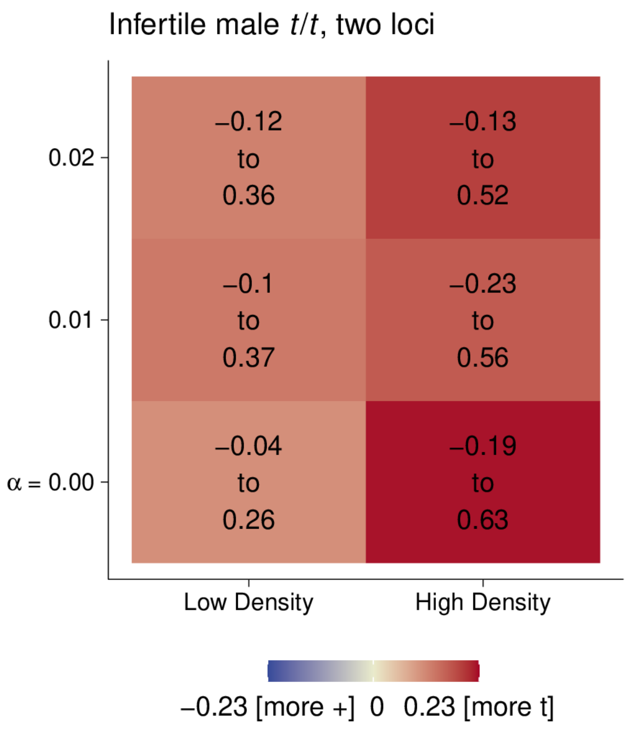

Suppl. Fig. 9: Heatmap showing the mean difference between t and + dispersal propensities in two-locus infertile male t/t models in low and high densities under varying polyandry α. Red indicates increased t dispersal, blue indicates increased + dispersal. The text indicates the 95% confidence interval.

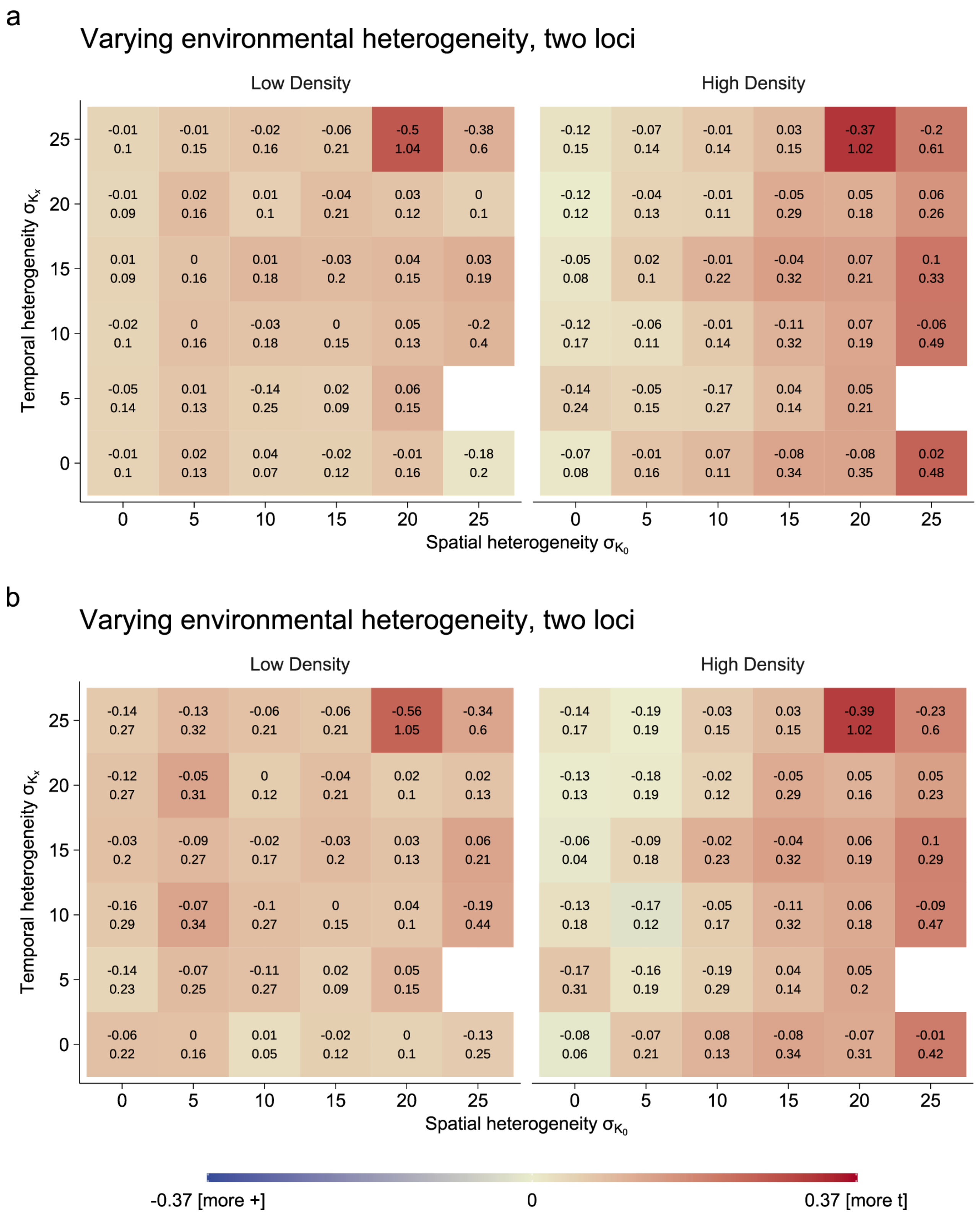

Suppl. Fig. 10: Heatmaps showing the mean difference between t and $+$ dispersal propensities in two-locus natural condition models in low and high densities under varying spatial and temporal carrying capacity heterogeneity. Red indicates increased t dispersal, blue indicates increased $+$ dispersal. White areas indicate that $+$ fixated, the text indicates the 95% confidence interval. **a)** What is considered high/low density is calculated based on the individual (x axis) σ_{K_0} levels. **b)** High/low density is based on $\sigma_{K_0} = 15$ like in the main text.
